# Unilateral Application of Cathodal tDCS Reduces Transcallosal Inhibition and Improves Visual Acuity in Amblyopic Patients

**DOI:** 10.3389/fnbeh.2018.00109

**Published:** 2018-05-29

**Authors:** Tommaso Bocci, Francesco Nasini, Matteo Caleo, Laura Restani, Davide Barloscio, Gianluca Ardolino, Alberto Priori, Lamberto Maffei, Marco Nardi, Ferdinando Sartucci

**Affiliations:** ^1^Section of Neurophysiopathology, Department of Clinical and Experimental Medicine, University of Pisa, Pisa, Italy; ^2^Clinical Center for Neurotechnologies, Neuromodulation, and Movement Disorders, Fondazione IRCCS Ca’Granda Ospedale Maggiore Policlinico, Milan, Italy; ^3^Department of Surgical, Medical, and Molecular Pathology and Critical Care, University of Pisa, Pisa, Italy; ^4^CNR Institute of Neuroscience, University of Pisa, Pisa, Italy; ^5^Department of Health Sciences, University of Milan and Ospedale San Paolo, Milan, Italy

**Keywords:** amblyopia, tDCS, amblyopia treatment in adults, corpus callosum, ocular dominance, visual system plasticity

## Abstract

**Objective:** Amblyopia is a neurodevelopmental disorder characterized by visual acuity and contrast sensitivity loss, refractory to pharmacological and optical treatments in adulthood. In animals, the corpus callosum (CC) contributes to suppression of visual responses of the amblyopic eye. To investigate the role of interhemispheric pathways in amblyopic patients, we studied the response of the visual cortex to transcranial Direct Current Stimulation (tDCS) applied over the primary visual area (V1) contralateral to the “lazy eye.”

**Methods:** Visual acuity (logMAR) was assessed before (T_0_), immediately after (T_1_) and 60’ following the application of cathodal tDCS (2.0 mA, 20’) in 12 amblyopic patients. At each time point, Visual Evoked Potentials (VEPs) triggered by grating stimuli of different contrasts (K90%, K20%) were recorded in both hemispheres and compared to those obtained in healthy volunteers.

**Results:** Cathodal tDCS improved visual acuity respect to baseline (*p* < 0.0001), whereas sham polarization had no significant effect. At T_1_, tDCS induced an inhibitory effect on VEPs amplitudes at all contrasts in the targeted side and a facilitation of responses in the hemisphere ipsilateral to the amblyopic eye; compared with controls, the facilitation persisted at T_2_ for high contrasts (K90%; Holm–Sidak *post hoc* method, *p* < 0.001), while the stimulated hemisphere recovered more quickly from inhibition (Holm–Sidak *post hoc* method, *p* < 0.001).

**Conclusions:** tDCS is a promising treatment for amblyopia in adults. The rapid recovery of excitability and the concurrent transcallosal disinhibition following perturbation of cortical activity may support a critical role of interhemispheric balance in the pathophysiology of amblyopia.

## Introduction

Amblyopia is a neurodevelopmental disorder clinically characterized by visual acuity and contrast sensitivity loss, refractory to pharmacological and mechanical treatments in adulthood (Holmes and Clarke, 2006): given the lack of any organic cause, it has been also defined as a disorder “in which the patient sees nothing and the doctor sees nothing” (Holmes and Clarke, 2006). Amblyopia results in an abnormal binocular experience due to a mismatch between the images perceived with each eye. Although the retina is generally spared, microscopic anatomical and structural abnormalities in lateral geniculate bodies and visual cortex can occur (von Noorden and Crawford, 1992; Davis et al., 2003); fMRI studies are consistent with the hypothesis of a selective involvement of the parvocellular stream at a precortical or early cortical site, thus leading to detection and processing deficit for high-contrast stimuli (Li et al., 2007; Hess et al., 2010).

Permanent monocular visual impairment is a risk for blindness, if the dominant eye is injured or becomes affected later in life (Williams et al., 2003). For this reason, the early treatment is critical. Eye-patching has been used for centuries, whereas the use of atropine has only recently emerged (Repka et al., 2005).

In the past few years, new approaches are being developed, such as dichoptic visual training aimed at stimulating the amblyopic eye, reducing the interocular suppression by balancing stimulus contrast between visual hemifields (Stewart et al., 2007; Vedamurthy et al., 2015b; Žiak et al., 2017). Nonetheless, all these treatments appear to be effective for up to 7 years of age (Holmes et al., 2011), showing transient and inconclusive results in older patients (Gao et al., 2018). Moreover, current treatments are often associated with residual monocular and binocular deficits (Pediatric Eye Disease Investigator Group et al., 2008), with a high rate of recurrence (Bhola et al., 2006).

In animal models, the corpus callosum (CC) plays a critical role in the suppression of deprived eye responses after a period of monocular occlusion (Restani et al., 2009; Cerri et al., 2010); in humans, callosal connections appear to inhibit the responsiveness of the neurons located in the opposite hemisphere (Bocci et al., 2014). Moreover, reduced visual cortex excitability has been observed in patients with amblyopia, possibly reflecting abnormally high levels of cortico-cortical inhibition (Thompson et al., 2008; Hess and Thompson, 2015). Thus, the reduced responses of the amblyopic eye may be due to active inhibition (suppression) within the primary visual cortex. Here we tested the contribution of interhemispheric pathways to such inhibition.

It has been recently proved that inter-hemispheric connections regulate cortical gain by dampening neural responses to high-contrast stimuli in the target hemisphere (Bocci et al., 2011). Concurrently, we have suggested that the rapid recovery of excitability and the persistent transcallosal disinhibition following perturbation of cortical activity may exert a key role in the pathophysiology of photosensitive epilepsy (Bocci et al., 2016). Altogether, we reasoned that changes in transcallosal inhibition may explain the unbalanced mechanisms of contrast gain control and ocular dominance in amblyopia. To this aim, we enrolled 12 patients and compared changes in visual acuity and Visual Evoked Potential (VEP) amplitudes induced by inhibitory cathodal transcranial Direct Current Stimulation (tDCS) applied to the occipital lobe contralateral to the amblyopic eye.

## Materials and Methods

### Participants and Experimental Protocol

Twelve adult patients with unilateral amblyopia (Table 1) and 12 sex and age-matched healthy volunteers were enrolled in the study (mean age 26.1 ± 6.0 years; range 24–44, five females). Patients had an intraocular acuity difference of at least 0.2 LogMAR and were classified as strabismic, anisometropic or mixed amblyopia (both strabismus and anisometropia; see Table 1). Anisometropia was defined as a spherical equivalent difference of 1 dioptre or more between the eyes. Best refractive correction was worn during testing. Healthy volunteers did not have history of neurological or psychiatric disorders and they were all drug-free. Controls with normal vision had 0.1 LogMAR acuity or better in each eye and no history of visual disorders.

**Table 1:** Demographic and clinical assessment.

Patient’s number	Previous treatment	Type of Amblyopia	Visual acuity (logMAR)	Timeline of intervention
1	None	LE	0.0	cathodal/sham
		RE Aniso	0.55 (0.55)	
2	None	LE	0.0	cathodal/sham
		RE Aniso	1.03 (1.00)	
3	None	RE	0.0	sham/cathodal
		LE Aniso	0.40 (0.38)	
4	None	RE	0.0	cathodal/sham
		LE Strab	1.03 (0.94)	
5	None	RE	0.0	sham/cathodal
		LE Aniso	0.22 (0.22)	
6	None	LE	0.0	cathodal/sham
		RE Strab	0.55 (0.58)	
7	Patching	LE	0.0	sham/cathodal
		RE Aniso	0.38 (0.42)	
8	None	RE	0.0	sham/cathodal
		LE Strab	0.40 (0.40)	
9	None	LE	0.0	sham/cathodal
		RE Aniso	1.00 (1.05)	
10	None	LE	0.0	cathodal/sham
		RE Mixed	0.83 (0.75)	
11	None	LE	0.0	cathodal/sham
		RE Aniso	0.92 (0.84)	
12	Patching	RE	0.0	sham/cathodal
		LE Strab	0.26 (0.26)	

*Each patient underwent both cathodal and sham tDCS, elapsed by at least 1 week. Visual acuity, expressed as LogMAR, refers to the first clinical evaluation, immediately after the enrollment, but it was checked again before the second treatment (either sham or cathodal; in this case, checked values of the “lazy eye” are expressed within brackets). *LE*, left eye; *RE*, right eye; *Aniso*, anisomoetropic amblyopia; *Strab*, strabismic amblyopia. LogMAR was calculated according to the formula: LogMAR = −log (decimal acuity)*.

In patients, visual acuity was assessed at baseline (T0), immediately after (T1) and 60’ following the completion of tDCS applied over the primary visual area (V1) contralateral to the amblyopic eye.

At same time points, VEPs were recorded both in amblyopic patients and controls, at two different luminance contrasts (K90% and K20%).

Patients were enrolled by a clinician (FN), whereas electrophysiological recordings were performed by a different neurologist (DB), both blinded to the tDCS condition.

Written informed consent was signed by all subjects prior to participation in the study, approved by the local ethical Committee in accordance with the tenets of Helsinki. The study was approved by the local ethical committee (registration number 3135), at the University of Pisa (formally named “Comitato Etico di Area Vasta Nord Ovest della Toscana”).

### Transcranial Direct Current Stimulation (tDCS)

We applied tDCS over the V1, using a battery-driven constant current stimulator (HDCStim, Newronika, Italy) and a pair of electrodes in two saline-soaked synthetic sponges with a surface area of 25 cm^2^ (5 × 5 cm). Amblyopic patients underwent both cathodal (real) and sham stimulation, while in healthy controls only the cathodal polarization was applied. For cathodal stimulation, the cathode was centered either on O1 or O2 (according to the 10–20 international EEG system) and the anode on the right shoulder.

Anatomical correspondence between the target region and V1 was confirmed by a navigated stimulation system (SofTaxic optically-tracked by EMS, Italy). Tridimensional space positions of the head and electrode were reproduced on the computer screen in relation to an average brain anatomy based on a 3D realistic MR-constructed brain model: in accordance with previous articles, the lower horizontal border of the electrode was marked by a scalp point superficial to the tentorium cerebelli, while the medial vertical one corresponded to a scalp point superficial to the brain location 1 cm lateral to the interhemispheric falx cerebri (Olma et al., 2013; Behrens et al., 2017).

Direct currents were applied for 20 min with an intensity of 1.5 mA (current density 0.06 mA/cm^2^). The intensity and duration of stimulation were comparable to those used in previous studies (Antal et al., 2004, 2006; Lang et al., 2007), below the threshold for tissue damage (Nitsche et al., 2003). tDCS strength remained below the sensory threshold throughout the experimental session. At the offset of tDCS, the current was decreased in a ramp-like manner, a method shown to achieve a good level of blinding among sessions (Gandiga et al., 2006; Galea et al., 2009). In the sham condition, the current was turned on for 5 s and then turned off in a ramp-shaped fashion.

### Visual Acuity Assessment

Each patient underwent a complete ophthalmologic examination to exclude other causes of poor vision, thus confirming that the patient’s refractive correction (where applicable) was accurate in order to perform the The Best-Corrected Visual Acuity (BCVA) testing.

The BCVA was tested for both eyes by means of standard “Early Treatment Diabetic Retinopathy Study (ETDRS) Revised” translucent visual acuity charts (with the following features: same number of letters per line, equal spacing between lines on a log scale, equal spacing of letters on a log scale and balanced letter difficulty in the individual lines). Retro-illuminated ETDRS viewing cabinet was used.

Both eyes were separately tested at a distance of 4 m (about 13 feet). Chart 1 was used for visual acuity testing of the right eye and chart 2 for testing the left eye. The patient was asked to read slowly, beginning from the top line of the chart, from left to right. The patient was told that one chance is given to read each letter. If the patient changed a response (e.g., ‘that was a “C” not an “O”’) before he/she has read the next letter, then the change was accepted.

- If a patient was able to read at least 20 letters on the chart, the visual acuity score of the tested eye was recorded as the number of letters read correctly at 4 m (sum = A) plus 30 (credit of 30 score points = B).
- If a patient could not read at least 20 letters on the chart at 4 m, the test was repeated at a distance of 1 m. In this case, the visual acuity score for the tested eye was recorded as the number of letters read correctly at 1 m (sum = C) plus the number of letters read correctly at 4 m (sum = A).

For each eye, the visual acuity score was the sum of A, B and C. If no letters were read correctly at either 4 m or 1 m, the visual acuity score was recorded as “0”. All procedures were done by an expert and certified ophthalmologist (FN).

### Visual Evoked Potentials (VEPs)

A detailed description of the protocol has been reported elsewhere (Bocci et al., 2011, 2016). VEPs were recorded in response to abrupt reversal (3 Hz) of a horizontal square wave grating (spatial frequency 2 c/deg), generated by computer on a display (Sony; refresh rate 60 Hz; subtending 20 × 15° of visual angle) by a VSG card (Cambridge Research Systems). The display was centered on the vertical meridian. VEPs were recorded simultaneously in both hemispheres, with Ag/AgCl electrodes positioned 2 cm above the inion (active) and at the right mastoid (reference).

VEP amplitudes were defined as the difference between the N1 negative peak and the P1 positive peak amplitudes in microvolts (Ding et al., 2016). The N1 was defined as a negative peak 60–110 ms after the pattern reversal and the peak of the first positive wave after N1 was named as P1.

VEPs were recorded before (T_0_), at the end (T_1_) and 45’ (T_2_) after tDCS. Grating stimuli were centered on the fixation point and tDCS was applied to V1. We analyzed 18 blocks of 100 averaged VEP responses (6 blocks at T0, 6 at T1 and 6 at T2), in terms of both mean amplitude (expressed as μV) and latency (ms) for two contrast levels (K90% and 20%). Visual stimuli at different contrasts were presented randomly and the obtained electrophysiological responses for each contrast were then averaged.

### Statistical Analysis

Parametric analyses were used, as all data sets successfully passed the Shapiro-Wilk test for normality (*p* > 0.05). A one-way analyses of variance (ANOVA) was used to compare baseline values for each subject between sham and cathodal condition. As VEP amplitudes are higher in healthy subjects and in the fellow eyes compared with the amblyopic ones, all values were normalized at baseline (T_0_, i.e., before tDCS: (T1/T0) × 100%).

#### Visual Acuity

In each patient, changes in visual acuity (logMAR) were assessed by using a two-way repeated-measures (RM) ANOVA, with “stimulation” (two levels: cathodal and sham) and “time” (three levels: T_0_, T_1_ and T_2_) as experimental factors, followed by Holm-Sidak *post hoc* method. The Pearson’s correlation was used to compare the average changes in visual acuity respect to the baseline values.

#### Electrophysiological Measures (VEPs)

A three-way repeated measures ANOVA assessed the effects of “time” × “stimulation” × “contrast” interaction in amblyopic patients. At each time interval, a two-way RM ANOVA compared peak-to-peak amplitudes between cathodal and sham polarization at different contrasts; significant effects were checked by *post hoc* Holm-Sidak test.

#### Comparison Between Amblyopic Patients and Controls

A three-way RM ANOVA analyzed the effects of “group” × “time” × “contrast” interaction between patients and healthy controls. At each time interval, a two-way ANOVA on ranks compared peak-to-peak amplitudes between amblyopic participants and healthy controls; significant effects were followed by *post hoc* Holm-Sidak test to compare VEP changes over time.

Statistical significance was set at *p* < 0.05. Data were analyzed using SPSS v. 21.0 for Windows (SPSS Inc., Chicago, IL, USA) or SigmaPlot v. 12.0.

## Results

### Clinical Assessment: Visual Acuity

Baseline (T_0_) logMAR values did not change between real and sham sessions (*p* = 0.83).

**Figure 1:**
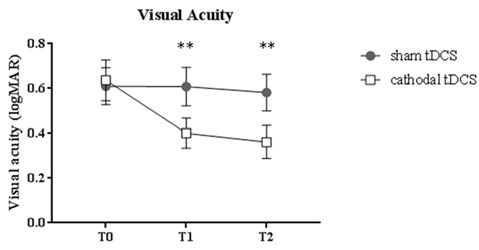
Changes in visual acuity (LogMAR). Amblyopic patients showed a significant improvement of visual acuity following cathodal transcranial direct current stimulation (tDCS) compared to sham polarization, with effects lasting for up to 1 h. Data are given as mean values ± standard error (SE); **p < 0.001.

A remarkable improvement occurred at T1 when cathodal polarization was delivered within the hemisphere contralateral to the amblyopic eye, with changes lasting up to 1 h after tDCS completion (*F*_(2,22)_ = 8.14, *p* = 0.0023, two-way ANOVA, with “time” and “treatment” as factors). This reduction ranged from 0.11 to 0.88 logMAR, with a mean of about 0.27 logMAR (see Table 2 and Figure 1), and it was significant both at T_1_ (*p* = 0.0029, Holm-Sidak *post hoc* comparison) and T_2_ (*p* = 0.0019) when compared to the sham group.

**Table 2:** Visual acuity following transcranial direct current stimulation (tDCS).

	Cathodal tDCS		Sham tDCS	
Patient’s number	T_1_	T_2_	T_1_	T_2_
1	−0.11	−0.33	−0.05	−0.11
2	−0.33	−0.47	0.03	−0.09
3	−0.14	−0.12	−0.02	−0.10
4	−0.61	−0.27	−0.19	−0.10
5	−0.07	−0.07	0.05	0.08
6	−0.22	−0.27	0.17	0.08
7	−0.22	−0.16	0.04	0.14
8	−0.25	−0.20	0.02	0.00
9	−0.35	−0.37	0.05	−0.06
10	−0.41	−0.08	−0.05	−0.07
11	−0.10	−0.88	0.07	0.08
12	−0.02	−0.08	0.02	−0.04

*Changes in logMAR score compared to baseline values are shown for the amblyopic eyes; notably, cathodal tDCS improved visual function both at T_1_ and T_2_*.

**Figure 2:**
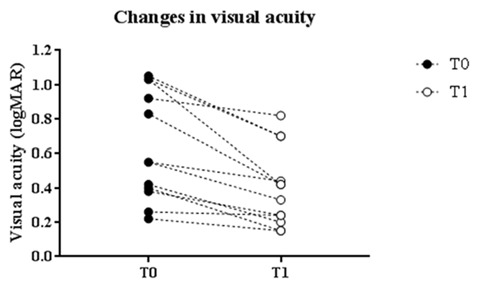
Correlation with baseline values. Changes in logMAR score linearly correlated with baseline values: patients with greater impairment at baseline showed a more robust enhancement of the visual acuity (*p* = 0.0004, Pearson’s correlation).

Changes in logMAR score linearly correlated with baseline values. Indeed, patients with greater impaired at baseline showed a more robust improvement in visual acuity (*p* = 0.0004, Pearson’s correlation; Figure 2).

### Visual Evoked Potentials (VEPs): Amblyopic Patients

At baseline, in agreement with previous data reported elsewhere (Ding et al., 2016), mean VEP amplitudes for amblyopic eyes were significantly lower than those recorded by stimulating the fellow eyes (6.54 ± 0.91 vs. 9.67 ± 1.99 μV at K90%: *t* = 4.61, *p* < 0.0001, unpaired *t*-test).

**Figure 3:**
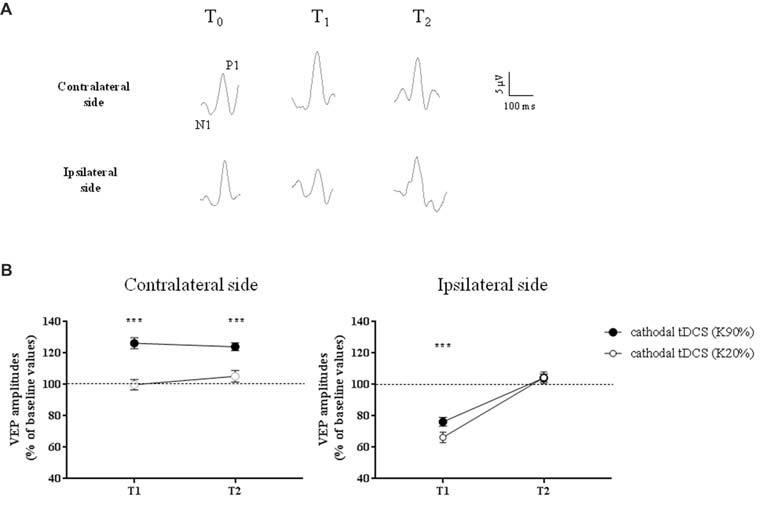
Changes in Visual Evoked Potential (VEP) amplitudes in amblyopic subjects. **(A)** Representative VEP responses to central stimulation (contrast, 90%) of the amblyopic eye, in the hemisphere contralateral (top traces) and ipsilateral (bottom traces) to tDCS intervention, respectively. **(B)** VEP amplitudes significantly increased ipsilaterally to the amblyopic eye, at high contrasts (K90%), and remained persistently elevated at T_2_ (*p* < 0.0001, Holm-Sidak *post hoc* test). On the opposite side, we observed a reduction of VEP amplitudes at T_1_, but at T_2_ all values returned to baseline, both for high (Holm-Sidak test, T_1_ vs. T_2_: *p* < 0.0001) and low contrasts (*p* = 0.001). ***p < 0.001.

Representative VEPs from one patient are shown in Figure 3A.

A three-way repeated measures ANOVA revealed significant effects of stimulation (*F*_(1,132)_ = 24.2, *p* < 0.0001), contrast (*F*_(1,132)_ = 96.8, *p* < 0.0001), stimulation × contrast (*F*_(1,132)_ = 23.3, *p* < 0.0001), time × stimulation (*F*_(2,132)_ = 8.7, *p* = 0.0003) and time × contrast interaction (*F*_(2,132)_ = 5.7, *p* = 0.0041). In particular, at high-contrast, VEP amplitudes recorded ipsilaterally to amblyopic eyes dramatically improved compared to low-contrast (*F*_(2,66)_ = 14.9, *p* < 0.0001, two-way ANOVA on ranks) and sham stimulation (*F*_(2,66)_ = 35.9, *p* < 0.0001, two-way RM ANOVA), remaining persistently elevated at T_2_ (*p* < 0.0001, Holm-Sidak test).

A significant correlation between the enhancement of visual acuity and the relative increase of VEP amplitudes in the amblyopic side was found (Pearson’s correlation: *p* = 0.002).

On the opposite side, as expected due to the inhibitory effect of cathodal polarization, we observed a reduction of VEP amplitudes at T_1_, both at high and low contrasts. At T_2_ all values returned to baseline, both for high (Holm-Sidak test, T_1_ vs. T_2_: *p* < 0.0001) and low contrasts (*p* = 0.001).

### Visual Evoked Potentials (VEPs): Comparison Between Patients and Controls

**Figure 4:**
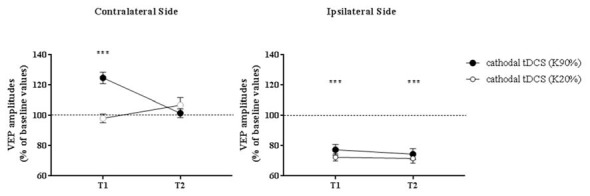
VEP amplitudes in subjects with normal visual acuity. VEP amplitudes increased on the side contralateral to the application of cathodal tDCS, while they were dampened ipsilaterally. Nonetheless, different from amblyopic participants, at T2 there was a loss of the contralateral facilitation, paralleled by a persistent inhibition of the responses recorded from the polarized hemisphere. Data are given as mean values ± standard error (SE); ***p < 0.0001.

When VEPs recorded from the side contralateral to tDCS were analyzed, a three-way ANOVA showed significant effects of time (*F*_(2,132)_ = 20.4, *p* < 0.0001), contrast (*F*_(1,132)_ = 64.9, *p* < 0.0001), time × contrast (*F*_(2,132)_ = 30.7, *p* < 0.0001), group × contrast (*F*_(1,132)_ = 11.4, *p* = 0.001) and contrast × group × time interaction (*F*_(2,132)_ = 8.0, *p* = 0.0005). When analyzed separately, at high contrasts (K90%), we found a persistent enhancement of VEP amplitude in amblyopic subjects but not controls at T_2_ (Holm-Sidak test, *p* < 0.0001): thus, transcallosal disinhibition persisted in amblyopic patients, while it vanished in controls (compare Figures 3, 4, contralateral side).

On the hemisphere in which inhibitory cathodal polarization was applied, patients and controls showed a similar reduction in VEP amplitudes at T1; a three-way ANOVA showed significant effects of time (*F*_(2,132)_ = 104.3, *p* < 0.0001), contrast (*F*_(2,132)_ = 3.9, *p* = 0.049) and group × time interaction (*F*_(2,132)_ = 49.8, *p* < 0.0001). At high contrasts, values returned to baseline in patients (Holm-Sidak test, T_1_ vs. T_2_ in patients, *p* < 0.0001), remaining significantly reduced in controls (Holm-Sidak test, T_1_ vs. T_2_ in controls, *p* = 0.42; compare Figures 3, 4).

## Discussion

Our results suggest that cathodal tDCS applied over the V1 contralateral to the “lazy eye” improves visual acuity, supporting the use of non-invasive brain stimulation techniques (NIBS) for the treatment of adult patients with amblyopia. Inhibitory cathodal tDCS dampened VEP amplitudes in both healthy and amblyopic subjects; concurrently, facilitation of visual responses in the contralateral side occurred, possibly due to the removal of interhemispheric inhibitory influences (Restani et al., 2009; Bocci et al., 2011). Significant differences were found at T_2_, with a faster normalization of VEP amplitudes in the stimulated side and a persistent disinhibition in the opposite hemisphere in amblyopic patients. This disinhibition may be at the basis of the behavioral improvement of visual acuity, which was detected at T1 and persisted at T2 (Figure 1). This interpretation is supported by the significant correlation between VEP changes and the enhancement of clinical outcome (i.e., reduction of logMAR).

A growing bulk of literature suggests that the adult visual system retains a high degree of plasticity (Lunghi et al., 2010, 2011; Lo Verde et al., 2017), indicating that the excitatory/inhibitory balance that modulates gain control mechanisms could be particularly susceptible to NIBS interventions, even at short timescales (Reinhart et al., 2016). In humans, previous articles have reported a significant effect of tDCS for the recovery of contrast sensitivity and stereopsis in amblyopia, providing a novel and safe approach to improve outcome in adults (Spiegel et al., 2013a; Ding et al., 2016). Authors demonstrated an enhancement of both monocular (visual acuity) and binocular (stereopsis) measures of visual function, especially when the polarization of the visual cortex was associated with dichoptic videogame-based treatment (Spiegel et al., 2013b). Nonetheless, these studies have used excitatory, anodal tDCS bilaterally applied over the V1. Here, we reasoned to dampen the excitability of the visual area contralateral to the “lazy eye”, with the aim to restore the balance of transcallosal inhibitory influences between hemispheres.

Our hypothesis is consistent with data in animals, showing that transcallosal connections are primarily involved in the weakening of deprived eye responses during monocular deprivation (Restani et al., 2009; Pietrasanta et al., 2014). Since transcallosal neurons are excitatory, interhemispheric inhibition depends upon the activation of GABAergic neurons in the target side, which contact local cortical pyramids via GABA-B receptors (Irlbacher et al., 2007; Palmer et al., 2012). Along this line, in primate models of amblyopia, the magnitude of side-to-side suppression seems to be closely related to the behavioral loss of contrast sensitivity in the amblyopic eye (Bi et al., 2011; Li et al., 2011, 2013; Tao et al., 2014).

Further support for a key role of interhemispheric pathways in amblyopia comes from recent data showing a higher vulnerability of the parvocellular pathway to the effects of visual deprivation, thus affecting the chromatic vision in humans (Hess et al., 2010; Lunghi et al., 2013). Notably, the callosum preferentially processes high-contrast stimuli and robustly transfers chromatic information related to the activation of the parvocellular stream (Berardi and Fiorentini, 1987; Berardi et al., 1987; Corballis, 1996; Roser and Corballis, 2003). Also in our sample, the persistent facilitation of visual responses has been observed for high contrasts only (see Figure 3).

### Limitations and Alternative Explanations

The main limitation of our study is the small number of patients, due to the difficulty in recruiting a homogeneous group of subjects. High-powered studies, with largest samples, are needed in the future to confirm our data and assess the efficacy of unilateral cathodal tDCS as a valuable option for the long-term treatment of amblyopia. Despite the low number of cases, cathodal tDCS displayed a consistent effect on visual acuity (see Figure 2).

Although our results appear to fit an explanation based on imbalance of V1 cortical excitability between hemispheres, additional possibilities need to be considered. First, the rapid changes triggered by tDCS (in terms of both visual acuity and VEP amplitudes) strongly support alterations in the excitatory/inhibitory balance within the visual system rather than structural rearrangements of inputs from the lazy eye. In this context, there is evidence that responses of the weak eye are actively suppressed by GABAergic inhibition (Duffy et al., 1976), and tDCS may alter GABA concentrations in the cerebral cortex (Stagg et al., 2009).

Second, the reduction of cortical excitability mediated by cathodal tDCS in the stimulated hemisphere could potentiate weak responses from the lazy eye via homeostatic mechanisms (Turrigiano, 2012). For example, it has been previously shown that brief period of monocular deprivation in adult subjects strongly alters ocular balance, producing a perceptual boost of the deprived eye (Lunghi et al., 2011). Along this line, the binocular imbalance that characterizes amblyopia can be reduced by occluding the amblyopic eye with a translucent patch for a few hours (Zhou et al., 2013).

Another possibility is that tDCS affects brainstem nuclei or thalamic structures, such as the lateral geniculate nucleus. In this case, the effects of the manipulation on acuity and VEP responses could be at least partly due to an action at subcortical level. Although this hypothesis cannot be definitely ruled out, VEP changes following hemifield visual stimulation seem to be consistent with a selective modulation of the interhemispheric route, as described in more detail elsewhere (Bocci et al., 2011, 2016). Moreover, direct geniculocortical connections are mildly affected by monocular deprivation in animals, with effects requiring at least 20 days of ocular deprivation (Antonini et al., 1999).

## Conclusion

Overall, our data support the use of unilateral cathodal tDCS for the treatment of amblyopia in adults, when pharmacological and mechanical therapies are completely ineffective; in order to improve and prolong the clinical outcome, both in adults and children, tDCS may be also combined with novel behavioral methods, comprising dichoptic training, perceptual learning and video gaming (Tsirlin et al., 2015; Vedamurthy et al., 2015a,b). Although promising, these therapies are currently influenced by visual attention, possibly narrowing their application in clinical practice.

## Author Contributions

TB: conception and design of research, data analysis, interpretation of the results, writing article; MC, LR, AP, MN and FS: conception and design of research, interpretation of the results, writing article; FN: data collection and analysis, patients’ enrollment; GA and LM: conception and design of research, interpretation of the results; DB performed the experiments, writing the article.

## Conflict of Interest Statement

The authors declare that the research was conducted in the absence of any commercial or financial relationships that could be construed as a potential conflict of interest.

## References

[B2] Antal A. Nitsche M. A. Kincses T. Z. Kruse W. Hoffmann K. P. Paulus W. (2004). Facilitation of visuo-motor learning by transcranial direct current stimulation of the motor and extrastriate visual areas in humans. Eur. J. Neurosci. 19, 2888–2892. 10.1111/j.1460-9568.2004.03367.x 15147322

[B1] Antal A. Nitsche M. A. Paulus W. (2006). Transcranial direct current stimulation and the visual cortex. Brain Res. Bull. 68, 459–463. 10.1016/j.brainresbull.2005.10.006 16459203

[B3] Antonini A. Fagiolini M. Stryker M. P. (1999). Anatomical correlates of functional plasticity in mouse visual cortex. J. Neurosci. 19, 4388–4406. 10.1523/JNEUROSCI.19-11-04388.1999 10341241 PMC2452998

[B4] Behrens J. R. Kraft A. Irlbacher K. Gerhardt H. Olma M. C. Brandt S. A. (2017). Long-lasting enhancement of visual perception with repetitive noninvasive transcranial direct current stimulation. Front. Cell. Neurosci. 11:238. 10.3389/fncel.2017.00238 28860969 PMC5559806

[B6] Berardi N. Bisti S. Maffei L. (1987). The transfer of visual information across the corpus callosum: spatial and temporal properties in the cat. J. Physiol. 384, 619–632. 10.1113/jphysiol.1987.sp016473 3656156 PMC1192281

[B5] Berardi N. Fiorentini A. (1987). Interhemispheric transfer of visual information in humans: spatial characteristics. J. Physiol. 384, 633–647. 10.1113/jphysiol.1987.sp016474 3656157 PMC1192282

[B7] Bhola R. Keech R. V. Kutschke P. Pfeifer W. Scott W. E. (2006). Recurrence of amblyopia after occlusion therapy. Ophthalmology 113, 2097–2100. 10.1016/j.ophtha.2006.04.034 17074568

[B8] Bi H. Zhang B. Tao X. Harwerth R. S. Smith E. L. III. Chino Y. M. (2011). Neuronal responses in visual area V2 (V2) of macaque monkeys with strabismic amblyopia. Cereb. Cortex 21, 2033–2045. 10.1093/cercor/bhq272 21263036 PMC3155601

[B9] Bocci T. Caleo M. Giorli E. Barloscio D. Maffei L. Rossi S.. (2011). Transcallosal inhibition dampens neural responses to high contrast stimuli in human visual cortex. Neuroscience 187, 43–51. 10.1016/j.neuroscience.2011.04.050 21557988

[B10] Bocci T. Caleo M. Restani L. Barloscio D. Rossi S. Sartucci F. (2016). Altered recovery from inhibitory repetitive transcranial magnetic stimulation (rTMS) in subjects with photosensitive epilepsy. Clin. Neurophysiol. 127, 3353–3361. 10.1016/j.clinph.2016.06.013 27407061

[B11] Bocci T. Pietrasanta M. Cerri C. Restani L. Caleo M. Sartucci F. (2014). Visual callosal connections: role in visual processing in health and disease. Rev. Neurosci. 25, 113–127. 10.1515/revneuro-2013-0025 24127537

[B12] Cerri C. Restani L. Caleo M. (2010). Callosal contribution to ocular dominance in rat primary visual cortex. Eur. J. Neurosci. 32, 1163–1169. 10.1111/j.1460-9568.2010.07363.x 20726891

[B13] Corballis M. C. (1996). A dissociation in naming digits and colors following commissurotomy. Cortex 32, 515–525. 10.1016/s0010-9452(96)80008-6 8886526

[B14] Davis A. R. Sloper J. J. Neveu M. M. Hogg C. R. Morgan M. J. Holder G. E. (2003). Electrophysiological and psychophysical differences between early- and late-onset strabismic amblyopia. Invest. Ophthalmol. Vis. Sci. 44, 610–617. 10.1167/iovs.02-0240 12556390

[B15] Ding Z. Li J. Spiegel D. P. Chen Z. Chan L. Luo G.. (2016). The effect of transcranial direct current stimulation on contrast sensitivity and visual evoked potential amplitude in adults with amblyopia. Sci. Rep. 6:19280. 10.1038/srep19280 26763954 PMC4725886

[B16] Duffy F. H. Burchfiel J. L. Conway J. L. (1976). Bicuculline reversal of deprivation amblyopia in the cat. Nature 260, 256–257. 10.1038/260256a0 1256565

[B17] Galea J. M. Jayaram G. Ajagbe L. Celnik P. (2009). Modulation of cerebellar excitability by polarity-specific noninvasive direct current stimulation. J. Neurosci. 29, 9115–9122. 10.1523/JNEUROSCI.2184-09.2009 19605648 PMC2760225

[B18] Gandiga P. C. Hummel F. C. Cohen L. G. (2006). Transcranial DC stimulation (tDCS): a tool for double-blind sham-controlled clinical studies in brain stimulation. Clin. Neurophysiol. 117, 845–850. 10.1016/j.clinph.2005.12.003 16427357

[B19] Gao T. Y. Guo C. X. Babu R. J. Black J. M. Bobier W. R. Chakraborty A.. (2018). Effectiveness of a binocular video game vs. placebo video game for improving visual functions in older children, teenagers, and adults with amblyopia: a randomized clinical trial. JAMA Ophthalmol. 136, 172–181. 10.1001/jamaophthalmol.2017.6090 29302694 PMC6584719

[B21] Hess R. F. Li X. Lu G. Thompson B. Hansen B. C. (2010). The contrast dependence of the cortical fMRI deficit in amblyopia; a selective loss at higher contrasts. Hum. Brain Mapp. 31, 1233–1248. 10.1002/hbm.20931 20063352 PMC6870632

[B20] Hess R. F. Thompson B. (2015). Amblyopia and the binocular approach to its therapy. Vision Res. 114, 4–16. 10.1016/j.visres.2015.02.009 25906685

[B22] Holmes J. M. Clarke M. P. (2006). Amblyopia. Lancet 367, 1343–1351. 10.1016/S0140-6736(06)68581-4 16631913

[B23] Holmes J. M. Lazar E. L. Melia B. M. Astle W. F. Dagi L. R. Donahue S. P.. (2011). Effect of age on response to amblyopia treatment in children. Arch. Ophthalmol. 129, 1451–1457. 10.1001/archophthalmol.2011.179 21746970 PMC3217111

[B100] Irlbacher K. Brocke J. Mechow J. V. Brandt S. A. (2007). Effects of GABA_A_ and GABA_B_ agonists on interhemispheric inhibition in man. Clin. Neurophysiol. 118, 308–316. 10.1016/j.clinph.2006.09.023 17174150

[B24] Lang N. Siebner H. R. Chadaide Z. Boros K. Nitsche M. A. Rothwell J. C.. (2007). Bidirectional modulation of primary visual cortex excitability: a combined tDCS and rTMS study. Invest. Ophthalmol. Vis. Sci. 48, 5782–5787. 10.1167/iovs.07-0706 18055832

[B27] Li X. Dumoulin S. O. Mansouri B. Hess R. F. (2007). Cortical deficits in human amblyopia: their regional distribution and their relationship to the contrast detection deficit. Invest. Ophthalmol. Vis. Sci. 48, 1575–1591. 10.1167/iovs.06-1021 17389487

[B25] Li J. Hess R. F. Chan L. Y. Deng D. Yang X. Chen X.. (2013). Quantitative measurement of interocular suppression in anisometropic amblyopia: a case-control study. Ophthalmology 120, 1672–1680. 10.1016/j.ophtha.2013.01.048 23622875

[B26] Li J. Thompson B. Lam C. S. Deng D. Chan L. Y. Maehara G.. (2011). The role of suppression in amblyopia. Invest. Ophthalmol. Vis. Sci. 52, 4169–4176. 10.1167/iovs.11-7233 21447685

[B28] Lo Verde L. Morrone M. C. Lunghi C. (2017). Early cross-modal plasticity in adults. J. Cogn. Neurosci. 29, 520–529. 10.1162/jocn_a_01067 27779909 PMC5289512

[B29] Lunghi C. Binda P. Morrone M. C. (2010). Touch disambiguates rivalrous perception at early stages of visual analysis. Curr. Biol. 20, R143–R144. 10.1016/j.cub.2009.12.015 20178754

[B30] Lunghi C. Burr D. C. Morrone C. (2011). Brief periods of monocular deprivation disrupt ocular balance in human adult visual cortex. Curr. Biol. 21, R538–R539. 10.1016/j.cub.2011.06.004 21783029

[B31] Lunghi C. Burr D. C. Morrone M. C. (2013). Long-term effects of monocular deprivation revealed with binocular rivalry gratings modulated in luminance and in color. J. Vis. 13:1. 10.1167/13.6.1 23637272

[B32] Nitsche M. A. Liebetanz D. Lang N. Antal A. Tergau F. Paulus W. (2003). Safety criteria for transcranial direct current stimulation (tDCS) in humans. Clin. Neurophysiol. 114, 2220–2222; author reply 2222–2223. 10.1016/s1388-2457(03)00235-9 14580622

[B33] Olma M. C. Dargie R. A. Behrens J. R. Kraft A. Irlbacher K. Fahle M.. (2013). Long-term effects of serial anodal tDCS on motion perception in subjects with occipital stroke measured in the unaffected visual hemifield. Front. Hum. Neurosci. 7:314. 10.3389/fnhum.2013.00314 23805097 PMC3690540

[B101] Palmer L. M. Schulz J. M. Murphy S. C. Ledergerber D. Murayama M. Larkum M. E. (2012). The cellular basis of GABA_B_-mediated interhemispheric inhibition. Science 335, 989–993. 10.1126/science.1217276 22363012

[B34] Pediatric Eye Disease Investigator Group Repka M. X. Kraker R. T. Beck R. W. Holmes J. M. Cotter S. A.. (2008). A randomized trial of atropine vs. patching for treatment of moderate amblyopia: follow-up at age 10 years. Arch. Ophthalmol. 126, 1039–1044. 10.1001/archopht.126.8.1039 18695096 PMC2614351

[B35] Pietrasanta M. Restani L. Cerri C. Olcese U. Medini P. Caleo M. (2014). A switch from inter-ocular to inter-hemispheric suppression following monocular deprivation in the rat visual cortex. Eur. J. Neurosci. 40, 2283–2292. 10.1111/ejn.12573 24689940

[B36] Reinhart R. M. Xiao W. McClenahan L. J. Woodman G. F. (2016). Electrical stimulation of visual cortex can immediately improve spatial vision. Curr. Biol. 26, 1867–1872. 10.1016/j.cub.2016.05.019 27374337 PMC4961578

[B37] Repka M. X. Wallace D. K. Beck R. W. Kraker R. T. Birch E. E. Cotter S. A.. (2005). Two-year follow-up of a 6-month randomized trial of atropine vs. patching for treatment of moderate amblyopia in children. Arch. Ophthalmol. 123, 149–157. 10.1001/archopht.123.2.149 15710809

[B38] Restani L. Cerri C. Pietrasanta M. Gianfranceschi L. Maffei L. Caleo M. (2009). Functional masking of deprived eye responses by callosal input during ocular dominance plasticity. Neuron 64, 707–718. 10.1016/j.neuron.2009.10.019 20005826

[B39] Roser M. Corballis M. C. (2003). Interhemispheric neural summation in the split brain: effects of stimulus colour and task. Neuropsychologia 41, 830–846. 10.1016/s0028-3932(02)00290-7 12631533

[B40] Spiegel D. P. Byblow W. D. Hess R. F. Thompson B. (2013a). Anodal transcranial direct current stimulation transiently improves contrast sensitivity and normalizes visual cortex activation in individuals with amblyopia. Neurorehabil. Neural Repair 27, 760–769. 10.1177/1545968313491006 23774122

[B41] Spiegel D. P. Li J. Hess R. F. Byblow W. D. Deng D. Yu M.. (2013b). Transcranial direct current stimulation enhances recovery of stereopsis in adults with amblyopia. Neurotherapeutics 10, 831–839. 10.1007/s13311-013-0200-y 23857313 PMC3805870

[B42] Stagg C. J. Best J. G. Stephenson M. C. O’Shea J. Wylezinska M. Kincses Z. T.. (2009). Polarity-sensitive modulation of cortical neurotransmitters by transcranial stimulation. J. Neurosci. 29, 5202–5206. 10.1523/JNEUROSCI.4432-08.2009 19386916 PMC6665468

[B43] Stewart C. E. Stephens D. A. Fielder A. R. Moseley M. J. MOTAS Cooperative (2007). Modeling dose-response in amblyopia: toward a child-specific treatment plan. Invest. Ophthalmol. Vis. Sci. 48, 2589–2594. 10.1167/iovs.05-1243 17525188

[B44] Tao X. Zhang B. Shen G. Wensveen J. Smith E. L. III. Nishimoto S.. (2014). Early monocular defocus disrupts the normal development of receptive-field structure in V2 neurons of macaque monkeys. J. Neurosci. 34, 13840–13854. 10.1523/JNEUROSCI.1992-14.2014 25297110 PMC4188977

[B45] Thompson B. Mansouri B. Koski L. Hess R. F. (2008). Brain plasticity in the adult: modulation of function in amblyopia with rTMS. Curr. Biol. 18, 1067–1071. 10.1016/j.cub.2008.06.052 18635353

[B46] Tsirlin I. Colpa L. Goltz H. C. Wong A. M. (2015). Behavioral training as new treatment for adult amblyopia: a meta-analysis and systematic review. Invest. Ophthalmol. Vis. Sci. 56, 4061–4075. 10.1167/iovs.15-16583 26114483

[B47] Turrigiano G. (2012). Homeostatic synaptic plasticity: local and global mechanisms for stabilizing neuronal function. Cold Spring Harb. Perspect. Biol. 4:a005736. 10.1101/cshperspect.a005736 22086977 PMC3249629

[B48] Vedamurthy I. Nahum M. Bavelier D. Levi D. M. (2015a). Mechanisms of recovery of visual function in adult amblyopia through a tailored action video game. Sci. Rep. 5:8482. 10.1038/srep08482 25719537 PMC4894407

[B49] Vedamurthy I. Nahum M. Huang S. J. Zheng F. Bayliss J. Bavelier D.. (2015b). A dichoptic custom-made action video game as a treatment for adult amblyopia. Vision Res. 114, 173–187. 10.1016/j.visres.2015.04.008 25917239 PMC4549206

[B50] von Noorden G. K. Crawford M. L. (1992). The lateral geniculate nucleus in human strabismic amblyopia. Invest. Ophthalmol. Vis. Sci. 33, 2729–2732. 1639619

[B51] Williams C. Northstone K. Harrad R. A. Sparrow J. M. Harvey I. ALSPAC Study Team. (2003). Amblyopia treatment outcomes after preschool screening v school entry screening: observational data from a prospective cohort study. Br. J. Ophthalmol. 87, 988–993. 10.1136/bjo.87.8.988 12881342 PMC1771818

[B52] Zhou J. Thompson B. Hess R. F. (2013). A new form of rapid binocular plasticity in adult with amblyopia. Sci. Rep. 3:2638. 10.1038/srep02638 24026421 PMC3770967

[B53] Žiak P. Holm A. Halicka J. Mojzis P. Piñero D. P. (2017). Amblyopia treatment of adults with dichoptic training using the virtual reality oculus rift head mounted display: preliminary results. BMC Ophthalmol. 17:105. 10.1186/s12886-017-0501-8 28659140 PMC5490155

